# Understanding the intersection of race and place: the case of tuberculosis in Michigan

**DOI:** 10.1186/s12889-019-8036-y

**Published:** 2019-12-11

**Authors:** Grace A. Noppert, Philippa Clarke, Margaret T. Hicken, Mark L. Wilson

**Affiliations:** 1 Carolina Population Center, University of North Carolina, 123 West Franklin St. Chapel Hill, Ann Arbor, NC 27516 USA; 2 Survey Research Center in the Institute for Social Research, University of Michigan, Ann Arbor, MI USA; 3 Department of Epidemiology, School of Public Health, University of Michigan, Ann Arbor, MI USA

**Keywords:** Social epidemiology, Health disparities, Race/ethnicity, Race and place, Urban health, Infectious disease epidemiology

## Abstract

**Background:**

Race and place intersect to produce location-based variation in disease distributions. We analyzed the geographic distribution of tuberculosis (TB) incidence in Michigan, USA to better understand the complex interplay between race and place, comparing patterns in Detroit, Wayne County and the state of Michigan as a whole.

**Methods:**

Using cross-sectional TB surveillance data from the Michigan Department of Health and Human Services, multivariable statistical models were developed to analyze the residence patterns of TB incidence from 2007 through 2012. Two-way interactions among the residence location and race of cases were assessed.

**Results:**

Overall, Detroit residents experienced 58% greater TB incidence than residents of Wayne County or the state of Michigan. Racial inequalities were less pronounced in Detroit compared to both Wayne County and the state of Michigan. Blacks in Detroit had 2.01 times greater TB incidence than Whites, while this inequality was 3.62 times more in Wayne County and 8.72 greater in the state of Michigan.

**Conclusion:**

Our results highlight how race and place interact to influence patterns of TB disease, and the ways in which this interaction is context dependent. TB elimination in the U.S. will require strategies that address the local social environment, as much as the physical environment.

## Background

Racial inequalities in both chronic and infectious diseases have been well-documented in the U.S. For example, in 2007 the mortality rate of Blacks was ~ 30% higher compared to Whites for 10 of the 15 leading causes of death [1]. These inequalities also hold true for infectious disease outcomes. For example, a 2009 study found that individuals of minority race/ethnicity status have a higher infectious burden than non-Hispanic Whites [2]. Despite widespread evidence of health inequalities by race in the U.S., the underlying determinants of these inequalities are not well-understood.

Racial group categories represent a complex set of interrelated historical processes that place people in separate and racially unequal contexts, with unequal social and economic exposures. This results in people of minority races often experiencing persistent disadvantage along a number of metrics, such as poor housing conditions, lack of access to material resources, and neighborhood environments that limit access to health care [3].

Given the contextual and relational nature of race, [4]. racial inequalities in health are then not static, but rather change over time and place as social and economic contexts change. With regard to place, for example, Black-White inequalities in mortality and life expectancy vary by region and local economic conditions. More specifically, the probability of survival of Black men from age 15 to age 65 in central Detroit in 1989–1991 was 50%, compared to 60% for White men. However, survival probability outside of central Detroit increased to 66 and 89% for Black and White men, respectively [5]. This general trend remained in 2000 when Black men in Eastside Detroit had 2.80 times the mortality rate of White men [5]. These data would suggest that racial inequalities may be reduced in areas with greater economic constraints experienced by both Black and White Americans. By the same argument, racial inequalities in health then may be larger in areas with more resources available for unequal distribution.

Tuberculosis (TB) presents an interesting case study in which to examine the intersection of race and place. Recent investigations have reported that at the neighborhood-level, socioeconomic disadvantage is positively associated with both incidence of TB, and increased incidence of recently transmitted TB [6–8]. Disadvantaged urban settings can enhance transmission of *Mycobacterium tuberculosis* (MTB) (the bacterium causing TB disease) through poverty, deteriorating housing conditions, social disorganization, reduced access to health care, and political disinvestment [9].

With a national TB annual incidence of 3.2 cases per 100,000 persons in 2012, [10] the U.S. is considered a low-burden country for TB disease. However, TB incidence has recently stagnated [11] after years of decline. Despite decades of organized TB control efforts, inequalities in TB disease persist, particularly along lines of social disadvantage [12, 13]. Previous studies have found that U.S.-born, non-Hispanic Blacks were the highest risk racial/ethnic group for TB incidence, [14] and that racial disparities were largely explained by markers of neighborhood disadvantage [15]. Moreover, consistent with other work on socioeconomic status (SES) in the U.S [16] there is an observable gradient in the distribution of TB, both at the individual- and neighborhood-level, whereby TB incidence is higher among those of lower SES [17–19].

The city of Detroit is the most populous in the state of Michigan. Located in southeastern Michigan’s Wayne County, Detroit has experienced dramatic socioeconomic and structural changes since the 1950s, including population decline, rising unemployment, infrastructural decay, and racial segregation [20]. Over a third of the city’s residents live beneath the poverty line. In 2010, 18% of Michigan’s population resided in Wayne County, including 7% in the city of Detroit [21] Yet, nearly 40% of the state’s TB cases from 2004 through 2012 occurred in Wayne County, most of which were in Detroit [14]. In the state of Michigan, TB patterns show considerable inequalities, particularly by race and nativity (place of birth) [14, 15]. However, it is unknown to what extent these race and nativity inequalities differ for Detroit compared to the rest of the state of Michigan.

Using six years of Michigan TB surveillance data (2007–2012), we analyzed socio-demographic risk factors for TB incidence among Detroit and Wayne County TB cases compared to those in the rest of the state of Michigan. We aimed to better understand how interactions of race/ethnicity and place produce TB disparities.

## Methods

### Case data

Since 1953, the U.S. Centers for Disease Control and Prevention (CDC) has mandated routine surveillance of TB across all states. We used surveillance data from the Michigan Department of Health and Human Services (MDHHS), including all TB cases diagnosed in the State of Michigan during January 1, 2007 through December 31, 2012. Cases with culture-confirmed TB disease, as well as those lacking culture confirmation but with clinical symptomology, were included in the analysis.

Demographic and clinical characteristics of all cases were drawn from de-identified data collected with the MDHHS “Report of a Verified Case of TB” form that was developed by the CDC. Race/ethnicity was coded as Non-Hispanic Black, Non-Hispanic White, Asian, and Hispanic. Gender and nativity were dichotomized as male or female and U.S.-born or foreign-born, respectively.

### Jurisdiction data

We used data from three separate jurisdictional areas: the state of Michigan, Wayne County (excluding Detroit City), and Detroit City. In 2012, the state of Michigan (excluding those from the Wayne County and Detroit City) had a population of approximately 8 million. The population (excluding Wayne County and Detroit City) was approximately 82% Non-Hispanic White, 8% Non-Hispanic Black, 2% Hispanic, and 5% Asian. Wayne County (excluding the city of Detroit), had a population of 1.1 million people of whom 76% were Non-Hispanic White, 13% Non-Hispanic Black, 4% Hispanic, and 4% Asian. Finally, Detroit City had a population of approximately 700,000 people. In Detroit City, 8.2% of the population was Non-Hispanic White, 81% Non-Hispanic Black, 7.5% Hispanic, and 1.1% Asian. Population data were derived from the American Community Survey 2012, 1-year estimates.

Because residential street address data were not available, we used the location of treatment to define the geographic location of cases. Treatment jurisdiction was determined by the address of the individual at the time of diagnosis, and for some this was temporary. For such individuals who were experiencing an unstable housing situation, the location of diagnosis (e.g. a hospital or clinic) was used to determine treatment jurisdiction. Thus, rather than an individual or clinician defining their jurisdiction, cases were assigned a jurisdiction based on treatment address. Detroit cases were those that received treatment at the Detroit City Health Department (29% of the total cases). Wayne County cases were those that received treatment at the Wayne County Health Department (13% of the total cases). All other cases in the database were considered outside of Detroit and Wayne (i.e. Michigan cases; 58% of the total cases).

### Statistical analyses

We fit multivariable, negative binomial regression models with a log link to examine the average incidence of TB by location, race, nativity, and gender. Negative binomial models allow estimation of the incidence rate, and are appropriate for count data when traditional Poisson regression models are overdispersed. Exponentiation of the beta coefficients permits estimation of the incidence rate ratios. Incidence rate ratios (IRRs) and 95% confidence intervals (CIs) were calculated in SAS version 9.4 (Carey, NC). For all analyses, a two-tailed alpha level of 0.05 was used to determine statistical significance.

The offset term in the regression model was the log of the total population. Population estimates were obtained using the Integrated Public Use Microdata Series (IPUMS) published by the University of Minnesota [22]. These data are derived from federal censuses of the U.S. population as well as American Community Surveys from 2007 to 2012 [21]. Estimates for the state of Michigan were calculated by subtracting the population estimates of Wayne County (including Detroit) from the estimates for the state as a whole. Estimates for Wayne County were calculated by subtracting the population estimates of Detroit City from the estimates for Wayne County as a whole.

Due to the well-established differences in TB risk by race, [7, 23] we tested the statistical significance of differences in the association between race and TB incidence across strata in the full sample. This included a cross-product interaction term in the negative binomial regression models to asses for multiplicative interaction between race and location. Models were then stratified by location to show differences in socio-demographic risk for TB in Detroit and Wayne County compared to the rest of the state of Michigan.

The study was approved by the Institutional Review Board for Health Sciences and Behavioral Sciences at the University of Michigan.

### Sensitivity analyses

In order to further contextualize our findings within larger trends in health and disease occurring in Detroit and the state of Michigan, we extracted data from the Behavioral Risk Factor Surveillance System (BRFSS). These data included the prevalence of a number of chronic conditions: chronic obstructive pulmonary disease (COPD), asthma, diabetes, coronary heart disease. We examined these trends by both race/ethnic category as well as age category.

## Results

From January 1, 2007 through December 31, 2012, a total of 1032 TB cases were reported in Michigan. Among those, 29% (*n* = 295) received treatment at the City of Detroit Health Department, 13% (*n* = 138) from the Wayne County Health Departments, and 58% (*n* = 599) received treatment elsewhere in Michigan. In Detroit, 81% of the cases were non-Hispanic Black compared to 17% of cases in Wayne County and 22% of cases in the rest of the state (Table 1). In contrast, 7% of the Detroit cases were Asian compared to 33% of the Wayne County cases and 34% of the cases in the rest of the state. Further, 85% of the Detroit cases were U.S.-born compared to only 38% of the Wayne County cases and 41% of the cases in the rest of Michigan.

**Table 1:** Distribution of Demographic and Clinical Risk Factors Among Michigan TB Cases 2007–2012 (*N* = 1032): Comparison of Cases in Detroit, those in Wayne County, and those in the rest of Michigan

Covariate	Detroit		Wayne County		Michigan	
	N	%	N	%	N	%
	295	29	138	13	599	58
Case Type						
Culture-confirmation	235	80	87	63	454	76
No culture-confirmation (clinical case)	60	20	51	37	145	24
Gender						
Male	185	63	80	58	350	58
Female	110	37	58	42	249	42
Race/Ethnicity						
Non-Hispanic Black	239	81	24	17	131	22
Non-Hispanic White	18	6	60	43	176	29
Asian	20	7	45	33	202	34
Hispanic	18	6	9	7	90	15
Nativity						
U.S.-born	251	85	52	38	243	41
Foreign-born	44	15	86	62	356	59
Site of Disease						
PTB	216	73	82	59	385	64
EPTB	55	19	41	30	152	25
Both PTB and EPTB	24	8	14	10	61	10
Missing	0		1	0.72	1	0.17
Age						
Median (SD) in years	49	21	52	22	45	23

EPTB = extrapulmonary TB; PTB = pulmonary TB

The Wayne County estimates are comprised of the number of cases in Wayne County minus the number of cases in Detroit City

The Michigan estimates are comprised of the number of cases in Michigan minus the cases in Wayne County and Detroit City

Individuals in Detroit had the largest burden of TB disease compared to those in Wayne County and the rest of the state of Michigan. The predicted annual incidence rate averaged across the six-year time period was highest in Detroit with a rate of 11 cases per 100,000 person (95% CI: 7.5, 16.1). The averaged predicted annual incidence rate was 5.5 cases per 100,000 persons (95% CI: 3.9, 7.8) for those in Wayne County and 9.4 cases per 100,000 persons (95% CI: 7.0, 12.6) for the state of Michigan as a whole. These trends are consistent with the incidence rate ratios estimated in Table 2. Adjusted Model 2 showed that those in Detroit had nearly twice the incidence rate as those in the rest of the state (IRR = 1.95, 95% CI: 1.56, 2.44). In Wayne County, there was a 13% lower TB incidence rate compared to the rest of Michigan (IRR = 0.87, 95% CI: 0.69, 1.08). Table 2 also presents the absolute numbers of TB cases for each subgroup.

**Table 2:** Incidence Rate Ratios for TB in Michigan 2007–2012 (N = 1032)

Covariate	N	Model 1		Model 2	
		IRR (95% CI)	*P* Value	IRR (95% CI)	P Value
Location					
Detroit	295	1.17 (0.72, 1.89)	0.02	1.95 (1.56, 2.44)	< 0.0001
Wayne County	135	0.58 (0.37, 0.92)		0.87 (0.69, 1.08)	
Michigan	599	Ref.		Ref.	
Race					
NH-Black	394			6.21 (4.83, 7.99)	< 0.0001
Asian	267			5.83 (4.45, 7.65)	
Hispanic	117			2.73 (2.05, 3.64)	
NH-White	254			Ref.	
Nativity					
Foreign-born	486			9.02 (7.34, 11.08)	< 0.0001
U.S.-born	546			Ref.	
Gender					
Male	615			1.50 (1.27, 1.79)	< 0.0001
Female	417			Ref.	

Models based on negative binomial regression with a log link

*P*-values reflect the two-sided Type 3 analysis effects

NH = Non-Hispanic; IRR = incidence rate ratio; CI = confidence interval

Furthermore, we found large racial inequalities in the incidence rate of TB (Table 2). Across the state of Michigan and independent of gender, location and nativity, non-Hispanic Blacks had an incidence rate 6.21 times that of non-Hispanic Whites. Similarly, Asians had 5.83 times and Hispanics 2.73 times that of non-Hispanic Whites (Table 2). A test for a two-way interaction between location and race/ethnicity was significant (*P < 0.0001*), indicating that we should stratify the subsequent analyses by location (data not shown).

Notably, we observed place-based variation in racial inequalities in TB. In the stratified analyses, we found that the inequalities for race/ethnicity were substantially smaller in Detroit compared to Wayne County and the rest of the state of Michigan (Table 3). Blacks in Detroit had 2.01 times (95% CI: 1.20, 3.37) the incidence rate of TB of Whites in Detroit. This Black-White inequality was less than the 3.62 times (95% CI: 2.18, 6.03) in Wayne County and 8.72 times (95% CI: 6.60, 11.53) in the state of Michigan.

**Table 3:** Incidence Rate Ratios for TB in Michigan (2007–2012): Stratified by Location (Detroit N = 1032)

Covariate	Detroit (N = 295)			Wayne County (*N* = 135)			Michigan (N = 599)		
	N	IRR (95% CI)	P Value	N	IRR (95% CI)	P Value	N	IRR (95% CI)	P Value
Nativity									
Foreign-born	44	3.37 (2.31, 6.02)	< 0.0001	86	12.41 (7.98, 19.29)	< 0.0001	356	10.26 (8.03, 13.12)	< 0.0001
U.S.-born	251	Ref.		52	Ref.		243	Ref.	
Race/Ethnicity									
NH-Black	239	2.01 (1.20, 3.37)	< 0.0001	24	3.62 (2.18, 6.03)	< 0.0001	131	8.72 (6.60, 11.53)	< 0.0001
Asian	20	4.29 (2.16, 8.53)		45	3.38 (2.08, 5.49)		202	7.00 (5.16, 9.52)	
Hispanic	18	0.81 (0.41, 1.60)		9	1.54 (0.74, 3.21)		90	4.32 (3.15, 5.92)	
NH-White	18	Ref.		60	Ref.		176	Ref.	
Gender									
Male	185	1.88 (1.43, 2.47)	0.0001	80	1.45 (1.0, 2.10)	0.05	350	1.41 (1.14, 1.73)	0.002
Female	110	Ref.		58	Ref.		249	Ref.	

Models based on the negative binomial regression with a log link

The 2-way interaction for race*nativity was not significant outside of Detroit

IRR = incidence rate ratio; CI: confidence interval

### Sensitivity analyses

Using the BRFSS, we examined trends in other chronic diseases by race/ethnicity and age category. Given the Detroit population is predominantly Black (81%) and the state of Michigan is predominantly White (82%), we used Black/White comparisons as a coarse approximation for comparing Detroit to the rest of the state of Michigan.

For Blacks, the highest proportion of COPD cases was among 45–64 year olds with a drop in the proportion among the 65+ age group (Additional file 1: Fig. S1). For Whites, the highest proportion of COPD cases was similarly among the 45–64 year olds, however, there was also a substantial portion of cases in the 65+ age group. A similar trend was seen for diabetes. This trend was more exaggerated when examining the distribution of coronary heart disease, stroke, and myocardial infarction. For all three of these conditions, the highest proportion of cases for Blacks was observed in the 45–64 year old group with much lower proportions in the 65+ age group. However, for Whites a considerably larger proportion of cases were observed in the 65+ range compared to the 45–64 year group. For asthma, the highest proportion of cases among Blacks was observed in the 25–44 age group compared to the 45–64 year old age group for Whites.

## Discussion

We sought to explore the intersection of race and place in the context of Michigan, and specifically Detroit using TB patterns as a case study. Our results have demonstrated that people living in Detroit had an average TB incidence nearly twice that of those in the state of Michigan. Moreover, our results suggest that inequalities in TB vary by place and that inequalities are smallest in Detroit, where White residents experience levels of disadvantage closer to that of racial/ethnic minorities. Race as a social construct has been a commonly held axiom of social scientists for decades. But the ways in which race is relationally constructed in a given space and a given historical and contemporary context and how that impacts the distribution of health and disease is still being unpacked. A substantial portion of TB risk in the U.S. occurs in urban settings [24]. Overall, 29% of Michigan TB cases occurred in Detroit City; 46% of the U.S.-born TB cases were diagnosed in Detroit City. Oren and colleagues estimated that 36% of U.S. incident TB occurs in 48 cities that account for only 15% of the U.S. population [24]. Similarly, the CDC estimates nearly 80% of TB cases occur in a metropolitan setting [25]. TB may be more likely to occur in urban settings because of properties of MTB transmission. Both the density of the population and the distribution and contact patterns of individuals within that space are key determinants of transmission likelihood when an infectious case is present [26].

However, our study found that the inequalities in TB burden between Detroit and the rest of Michigan were not explained by population density. In the null model with only location of diagnosis as the predictor, there was no association between being a Detroit City resident and risk of TB. The statistical models control for population size with the offset term; thus, in the null model there was no additional risk for people in Detroit net of population size. There was an association between living in Detroit and TB risk when race, nativity, and gender were included in the model, suggesting confounding by socio-demographic characteristics not explained simply by a greater proportion of the population residing there. These findings underscore the influence of the social environment on the distribution of TB. In conjunction with the findings that the racial inequalities differed across each of the three locations (Detroit City, Wayne County, and Michigan), our study highlights how the ways in which the social environment influences the distribution of TB is context dependent. In a study comparing Black and White mortality rates across 16 different disadvantaged areas in the U.S., Geronimus et al. found the mortality inequality between advantaged and disadvantaged groups differed by geographic region. For example, while in most cases disadvantaged Blacks had more excess mortality than disadvantaged Whites, in Detroit white residents had mortality rates comparable to some Black populations studied [5]. Consistent with this finding, in multivariable models we observed a reduction in disparities by race and nativity within Detroit compared to cases in Wayne County and the rest of the Michigan.

These findings also suggest that the core of the inequality in TB disease lies in comparing Detroit to the rest of Michigan. Detroit residents have higher rates of social disadvantage across a number of metrics: higher proportion of individuals with less than a high school education, higher unemployment rate, lower per capita income, and a higher proportion of the population living in poverty, particularly among those under 18 years old and across racial/ethnic groups [21]. This increased social disadvantage has consequences for a number of health conditions, including TB.

There are several hypothesized mechanisms for how such disadvantage works to produce the disparities observed in TB disease. The first pathway operates through comprised immunity either through prolonged exposure to psychosocial stress and/or increased prevalence of co-morbid conditions. Prolonged experiences of social disadvantage results in increased exposure to psychosocial stress. This stress exposure leads to physiological wear and tear across a number of body systems, and specifically of interest to infectious disease researchers are the effects on the immune system. Chronic stress resultant from low SES is linked to increased inflammation [27, 28] and decreased immune function [29, 30]. Similarly, co-morbid health conditions often have negative consequences for immune function. The population of Detroit overall has a higher prevalence of diabetes, body mass index greater or equal to 30, and blood pressure compared to the State of Michigan, [31] all of which have known consequences for immune function. The hypothesis regarding the consequences of co-morbidities for TB disease can further be supported by examining age patterns in both TB disease and chronic conditions. Given that that the majority of TB cases among Black individuals occurred in Detroit, we used data from the BRFSS comparing Blacks to White in Michigan to better understand this relationship. For a number of chronic conditions, the Black/White disparity is greatest among those aged 25–44 years and those aged 45–64 years [31]. For example, Blacks have a higher prevalence than Whites of COPD, asthma, diabetes, coronary heart disease, and the prevalence peaks in younger age groups [31]. This is consistent with the trends we observed in TB in which Blacks had a higher prevalence of TB in ages 25–44 and 45–64 years whereas Whites had a higher prevalence in age 65 years and older. While we do not have the data to directly test this hypothesis, these two separate data points lend support that exposure to higher level of chronic conditions may be part of the constellation of social conditions making individuals more vulnerable to TB disease in Detroit.

The second hypothesized mechanism explaining the link between social disadvantage and TB disease is an indirect pathway through increased exposure to the pathogen causing TB disease, MTB. Using a fundamental cause of disease frame, the high levels of social disadvantage in Detroit results in poor housing conditions, lack of access to health care (including timely diagnostic services), lack of material resources (such as nutrition), and poor neighborhood environments [3, 9]. Considering both pathways, individuals in Detroit are both more likely to be exposed to MTB and more likely to progress to active infection once exposed.

Our goal in this investigation was to understand how the intersection of race and place in Michigan impacts inequalities in TB incidence. The findings from this study demonstrate the ways in which the Detroit setting is fundamentally different than the rest of Michigan, and the complex interaction between race/ethnicity and location must be investigated separately. The political and economic disinvestment that has characterized Detroit over the past few decades affects the entire population—producing worse health overall. The Detroit disparity is also inextricably linked to racial/ethnic disparities. The concentration of TB cases among Black individuals in Detroit reflects macro-level Black/White segregation occurring in Michigan [32].

### Strengths and limitations

This is one of the first studies to examine social disparities in TB comparing Detroit to the rest of Michigan. The use of state-level surveillance data, including both culture-confirmed cases and those with a clinical diagnosis, is a strength of our analysis. However, our designation of cases residing in or outside of the Detroit area was based on the health department delivering treatment to the individual, which was then used as proxy for residence. This assumption may not be true for some people, such as those living in homeless shelters or near the Detroit city limits. However, the coarse spatial designation that we employed (i.e., county) should minimize any misclassification that could have occurred, as most individuals likely received treatment in their county of residence, regardless of their residential address.

We analyzed all diagnosed TB cases in Michigan during the study time period, including some without culture-confirmation. Since our goal was to understand the broad population dynamics of TB in Michigan, excluding cases without culture-confirmation might systemically exclude certain populations. However, the limitation in such an approach is that some individuals without culture-confirmation may not have had TB disease, but this would seem to be extremely rare. In addition, these data represent trends through 2012. While analyses with more recent data would certainly be useful, we believe the trends observed in the current study are still generalizable to trends in Michigan in the present period.

Our findings highlight interesting and important trends with regard to the Asian population. However, such a coarse designation ignores important differences among ethnic categories within this classification. We lacked any data to disaggregate the Asian category further; future studies would benefit from a more thorough examination of this racial/ethnic group. Finally, because the population denominators were estimates based on the ACS data, which did not include age, we were unable to examine age patterns in our analyses.

## Conclusions

We found that how race/ethnicity and place interact to influence patterns of TB disease is dependent on the context in which this interaction occurs. Indeed, our findings lend further support to the notion that race is not static, it is socially and relationally constructed based on both the historical and contemporary context. Thus, understanding contemporary drivers of TB inequalities requires an acknowledgement of the historical and modern roots of structural inequality. TB elimination in the U.S. will demand strategies that can address the local social environment, [33] as much as the physical environment.

## Supplementary information

**Additional file 1 Figure S1**. Distribution of chronic conditions among Blacks and Whites in Michigan, stratified by age group using the Behavioral Risk Factor Surveillance System, 2012.

## Data Availability

The data analyzed in this study were made available to us from the Michigan Department of Health and Human Services under a data use agreement. Although these data are not publicly available, they may be obtained through request to the Michigan Department of Health and Human Services.

## Abbreviations

ACS: American Community Survey
CDC: Centers for Disease Control and Prevention
CI: Confidence interval
IRR: Incidence rate ratio
LTBI: Latent TB infection
MDHHS: Michigan Department of Health and Human Services
MTB: *Mycobacterium tuberculosis*
SES: Socioeconomic status
TB: Tuberculosis

## References

[1] Williams DR (2012). Miles to go before we sleep: racial inequities in health. J Health Soc Behav.

[2] Zajacova A, Dowd JB, Aiello AE (2009). Socioeconomic and race/ethnic patterns in persistent infection burden among U.S. adults. J Gerontol A Biol Sci Med Sci.

[3] Link BG, Phelan JC (1996). Understanding sociodemographic differences in health--the role of fundamental social causes. Am J Public Health.

[4] Geronimus AT (2000). To mitigate, resist, or undo: addressing structural influences on the health of urban populations. Am J Public Health.

[5] Geronimus AT, Bound J, Colen CG (2011). Excess black mortality in the United States and in selected black and white high-poverty areas, 1980–2000. Am J Public Health.

[6] Oren E, Koepsell T, Leroux B, Mayer J (2012). Area-based socio-economic disadvantage and tuberculosis incidence. Int J Tuberculosis Lung Disease.

[7] Oren E, Narita M, Nolan C, Mayer J (2014). Neighborhood socioeconomic position and tuberculosis transmission: a retrospective cohort study. BMC Infect Dis.

[8] Oren E, Narita M, Nolan C, Mayer J (2013). Area-level socioeconomic disadvantage and severe pulmonary tuberculosis: US, 2000–2008. Public Health Rep.

[9] Acevedo-Garcia D (2000). Residential segregation and the epidemiology of infectious diseases. Soc Sci Med.

[10] Control CfD, Prevention. Trends in tuberculosis--United States, 2012. *MMWR Morbidity and mortality weekly report.* 2013;62(11):201. PMC4604910 23515056

[11] Salinas Jorge L., Mindra Godwin, Haddad Maryam B., Pratt Robert, Price Sandy F., Langer Adam J. (2016). Leveling of Tuberculosis Incidence — United States, 2013–2015. MMWR. Morbidity and Mortality Weekly Report.

[12] Driver C, Kreiswirth B, Macaraig M (2007). Molecular epidemiology of tuberculosis after declining incidence, New York City, 2001–2003. Epidemiology & Infection.

[13] Alami NN, Yuen CM, Miramontes R, Pratt R, Price SF, Navin TR (2014). Trends in tuberculosis—United States, 2013. MMWR Morb Mortal Wkly Rep.

[14] Noppert GA, Wilson ML, Clarke P, Ye W, Davidson P, Yang Z (2017). Race and nativity are major determinants of tuberculosis in the US: evidence of health disparities in tuberculosis incidence in Michigan, 2004–2012. BMC Public Health.

[15] Noppert Grace A., Yang Zhenhua, Clarke Philippa, Ye Wen, Davidson Peter, Wilson Mark L. (2017). Individual- and neighborhood-level contextual factors are associated with Mycobacterium tuberculosis transmission: genotypic clustering of cases in Michigan, 2004–2012. Annals of Epidemiology.

[16] Marmot MG, Shipley MJ, Rose G (1984). Inequalities in death—specific explanations of a general pattern?. Lancet.

[17] Olson NA, Davidow AL, Winston CA, Chen MP, Gazmararian JA, Katz DJ (2012). A national study of socioeconomic status and tuberculosis rates by country of birth, United States, 1996–2005. BMC Public Health.

[18] Davidow AL, Mangura BT, Napolitano EC, Reichman LB (2003). Rethinking the socioeconomics and geography of tuberculosis among foreign-born residents of New Jersey, 1994–1999. Am J Public Health.

[19] Cantwell MF, McKENNA MT, McCRAY E, Onorato IM (1998). Tuberculosis and race/ethnicity in the United States: impact of socioeconomic status. Am J Respir Crit Care Med.

[20] Sugrue TJ. *The Origins of the Urban Crisis: Race and Inequality in Postwar Detroit-Updated Edition.* Vol 6: Princeton University Press; 2014.

[21] American Community Survey 2012 (5-Year Estimates) Social Explorer Tables: ACS 2012 (5-Year Estimates) (SE). . In: Office USCsACS, ed2012.

[22] S R, K G, R G, al. e. IMPUS 2017. In: 7.0 IUV, ed. Minneapolis, MN 2017.

[23] Rodwell TC, Kapasi AJ, Barnes RF, Moser KS (2012). Factors associated with genotype clustering of mycobacterium tuberculosis isolates in an ethnically diverse region of southern California, United States. Infect Genet Evol.

[24] Oren E, Winston CA, Pratt R, Robison VA, Narita M (2011). Epidemiology of urban tuberculosis in the United States, 2000–2007. Am J Public Health.

[25] Scott C, Kirking HL, Jeffries C, Price SF, Pratt R (2015). Tuberculosis trends—United States, 2014. MMWR Morb Mortal Wkly Rep.

[26] Giesecke J. Modern infectious disease epidemiology: CRC Press; 2017.

[27] Pollitt R, Kaufman J, Rose K, Diez-Roux AV, Zeng D, Heiss G (2008). Cumulative life course and adult socioeconomic status and markers of inflammation in adulthood. J Epidemiol Community Health.

[28] Friedman EM, Herd P (2010). Income, education, and inflammation: differential associations in a national probability sample (the MIDUS study). Psychosom Med.

[29] Fagundes CP, Bennett JM, Alfano CM (2012). Social support and socioeconomic status interact to predict Epstein-Barr virus latency in women awaiting diagnosis or newly diagnosed with breast cancer. Health Psychol.

[30] Janicki-Deverts D, Cohen S, Doyle WJ, Marsland AL, Bosch J (2014). Childhood environments and cytomegalovirus serostatus and reactivation in adults. Brain Behav Immun.

[31] Behavioral Risk Factor Surveillance System Survey Data In: Prevention CfDCa, ed2012.

[32] Lichter DT, Parisi D, Taquino MC (2015). Toward a new macro-segregation? Decomposing segregation within and between metropolitan cities and suburbs. Am Sociol Rev.

[33] Theron G, Jenkins HE, Cobelens F (2015). Data for action: collection and use of local data to end tuberculosis. Lancet.

