# Supplementary material for: Understanding the intersection of race and place: the case of tuberculosis in Michigan
Source: BMC Public Health. 2019 Dec 11;19:1669. doi: 10.1186/s12889-019-8036-y (PMC6907243; doi:10.1186/s12889-019-8036-y)
Supplement: Supplementary file 1 — Additional file 1 Figure S1. Distribution of chronic conditions among Blacks and Whites in Michigan, stratified by age group using the Behavioral Risk Factor Surveillance System, 2012. [file 12889_2019_8036_MOESM1_ESM.docx]

**Supplementary Figure 1**. Distribution of chronic conditions among Blacks and Whites in Michigan, stratified by age group using the Behavioral Risk Factor Surveillance System, 2012.

1A. Distribution of Chronic Obstructive Pulmonary Disease (COPD) 1B. Distribution of Diabetes

1C. Distribution of Coronary Heart Disease or Angina 1D. Distribution of Stroke

1E. Distribution of Myocardial Infarction 1F. Distribution of Asthma
